# Causal relationship between dried fruit intake and meniscal injuries: Two-sample Mendelian randomization

**DOI:** 10.1097/MD.0000000000036415

**Published:** 2023-12-01

**Authors:** Guang-Hua Deng

**Affiliations:** a Ya’an Hospital of Traditional Chinese Medicine.

**Keywords:** dried fruits, intake, Mendelian randomization, meniscus injury

## Abstract

To investigate the causal relationship between dried fruit intake and meniscal injuries using Mendelian randomization (MR). Data were pooled from large-scale genome wide association studies (GWAS), and genetic loci independently associated with dry fruit intake and meniscal injuries in populations of European origin were selected as instrumental variables. Three MR analyses, inverse variance weighting (IVW), weighted median (WME) and MR-Egger, were used to investigate the causal relationship between dried fruit intake and meniscal injuries. The results were tested for robustness by heterogeneity and multiplicity tests, and sensitivity analyses were performed using the “leave-one-out” method. The IVW results showed an OR (95 % CI) of 0.47 (0.28–0.78), *P* = .003, indicating a causal relationship between dried fruit intake and meniscus injury. And no heterogeneity and multiplicity were found by the test and sensitivity analysis also showed robust results. The present study used a 2-sample MR analysis, and by analyzing and exploring the genetic data, the study showed that too little intake of dry fruits is a risk factor for meniscal injuries.

## 1. Introduction

The meniscus is an important component within the knee joint, which stabilizes the knee joint, transmits knee loading forces and promotes intra-articular nutrition.^[1]^ It is the load stabilizing role played by the meniscus that ensures that the knee joint is protected from injury over years of weight-bearing exercise.^[2]^ Meniscus injury is an injury or tear to the meniscus tissue on the medial and lateral sides of the knee joint.^[3]^ Meniscus injuries are usually caused by strenuous torsional or rotational movements, direct impact on the knee, or overuse.^[4]^ In recent years, many studies have found that dried fruit intake is associated with the development of a variety of diseases, such as too little intake of dried fruits increases the incidence of cardiovascular disease,^[5]^ and too little intake of dried fruits also increases the incidence of asthma^[6]^ and low back pain.^[7]^ However, studies on dried fruit intake and meniscal injuries are lacking and the causal relationship is uncertain. The causal relationship between dried fruit intake and meniscal injuries still needs further investigation.

Mendelian randomization (MR), a genetic epidemiological method, is a useful tool for assessing the causal role of dried fruit intake and meniscal injuries.^[8]^ By using genetic variants such as single nucleotide polymorphism (SNP) as instrumental variants that can modify disease risk factors or exposures, MR studies can enhance causal inference of exposure-outcome associations.^[9]^ According to Mendel laws of inheritance, genetic variants are not susceptible to confounding factors because they are randomly assigned during gamete formation.^[10]^ In addition, confounders and reverse causation can be minimized as genotypes cannot change as the disease progresses.^[11]^

To this end, we conducted a 2-sample MR study to examine the association of dried fruit intake with causality in meniscal injuries. We aimed to provide significant evidence for the causal role of dried fruit intake in causing meniscal injuries.

## 2. Data and methods

### 2.1. Data sources

GWAS data on dried fruit intake and meniscal injuries were obtained via the IEU OpenGWAS project (mr cieu.ac.uk) website. The website was accessed on 2023-09-22, and all data ultimately obtained in this study were from a European population. Including dried fruit intake (ukb-b-16576) containing 9851,867 SNPs with a sample size of 421,764 individuals, and meniscal injuries (finn-b-M13_MENISCUSDERANGEMENTS) containing 16,380,200 SNPs with 13,568 individuals in the trial group and 147,221 individuals in the control group. This study was a re-analysis of previously collected and published publicly available data and therefore did not require additional ethical approval.

### 2.2. Conditioning of SNP as an instrumental variable

Firstly, the instrumental variables were highly correlated with exposure, with F > 10 as a strong correlation criterion.^[12]^ Secondly, the instrumental variable is not directly related to the outcome, but only affects the outcome through exposure, that is, there is no genetic pleiotropy. In this study, the nonexistence of genetic pleiotropy was indicated by a non-zero intercept term (*P* < .05) in the MR-Egger regression model.^[13]^ Finally, instrumental variables were not associated with untested confounding.^[14]^ The human genotype-phenotype association database Phenoscanner V2 was searched for phenotypes associated with the instrumental variables at the genome-wide significance level to determine whether these SNPs were associated with potential risk factors.^[15]^

### 2.3. SNP screening

Significant SNPs were screened from the GWAS pooled data of dried fruit intake (*P* < 5 × 10- 8 was used as the screening condition)^[16]^; the linkage disequilibrium coefficient r2 was set to be 0.001 and the width of the linkage disequilibrium region to be 10,000 kb to ensure that the individual SNPs were independent of each other.^[17]^ The SNPs related to dried fruit intake screened above were extracted from the GWAS pooled data of meniscus injury, while SNPs directly related to outcome indicators were excluded (*P* < 5 × 10- 8). The F-value of each SNP was calculated, and SNPs with weak instrumental variables (F-value <10) were excluded.^[18]^ And the human genotype-phenotype association database was queried to screen for potentially relevant risk factor SNPs and exclude them.^[19]^

### 2.4. Causality validation methods

The causal relationship between exposure (dried fruit intake) and outcome (meniscus injury) was mainly verified using inverse variance weighted (IVW) as, supplemented by MR-Egger and Weighted median (WME) MR analyses, with SNPs as instrumental variables.

### 2.5. Sensitivity analysis

Sensitivity analyses were performed using several methods. First, the Cochran Q test was used to assess the heterogeneity among the individual SNP estimates, and a statistically significant Cochran Q test proved that the analyses were significantly heterogeneous. Second, Mendelian randomization pleiotropy residual sum and outlier (MR PRESSO) was used to validate the results in the IVW model, to correct for the effect of outliers, and if outliers existed, they were removed and the analysis was repeated. Third, the horizontal multiplicity of SNPs was tested using the MR Egger intercept test (MR Egger intercept test), and if the intercept term in the MR Egger intercept test analysis was statistically significant, it indicated that the MR analysis had significant horizontal multiplicity. Fourth, “leave-one-out” sensitivity analyses were performed by removing a single SNP at a time to assess whether the variant drove the association between the exposure and outcome variables. Fifth, funnel plots and forest plots were constructed to visualize the results of the sensitivity analyses. *P* < .05 suggests that there is a potential causal relationship in the MR analyses, which is statistically significant. All statistical analyses were performed using the “TwoSampleMR” package in R software version 4.3.0.

## 3. Results

### 3.1. Instrumental variables

Forty-three SNPs that were strongly associated with dried fruit intake (*P* < 5 × 10- 8) without linkage disequilibrium (r2 < 0.001, kb = 10,000) were screened in the current study. Forty-three SNPs were left by taking the intersection with SNPs in the pooled data from the GWAS for meniscal injuries, and also by eliminating SNPs that were directly associated with the outcome metrics. In our study, the F-value of each SNP was >10, indicating no weak instrumental variables (Table 1). We searched the human genotype-phenotype association database and found no potentially relevant risk factor SNPs.

**Table 1:** Information on the final screening of dried fruit intake SNPs from GWAS data (n = 43).

ID	SNP	Effect_Allele	Other_Allele	β	SE	*P*	F
1	rs10026792	A	G	0.0108465	0.0018423	3.90E-09	34
2	rs10129747	G	A	0.00935902	0.00168126	2.60E-08	30
3	rs10740991	C	G	0.016739	0.00185722	2.00E-19	81
4	rs10896126	G	A	−0.015009	0.00181919	1.60E-16	68
5	rs11037497	C	G	0.0104396	0.00168442	5.70E-10	38
6	rs11152349	A	G	0.00991215	0.0018176	4.90E-08	29
7	rs11586016	C	G	0.00987818	0.00173034	1.10E-08	32
8	rs11632215	C	A	−0.0141434	0.00258382	4.40E-08	29
9	rs11720884	G	A	0.0111797	0.00193554	7.60E-09	33
10	rs11772627	C	G	0.0183338	0.00217099	3.00E-17	71
11	rs11811826	A	T	0.0132178	0.0020058	4.40E-11	43
12	rs12137234	T	C	0.0102051	0.00183744	2.80E-08	30
13	rs1582322	G	A	0.00994346	0.00171571	6.80E-09	33
14	rs1622515	G	A	0.00991719	0.00167077	2.90E-09	35
15	rs1648404	T	C	0.00941595	0.00167357	1.80E-08	31
16	rs17175518	A	C	0.0114962	0.00197514	5.90E-09	33
17	rs17184707	T	C	−0.0114381	0.00204011	2.10E-08	31
18	rs1797235	C	G	−0.0100206	0.00174235	8.90E-09	33
19	rs2328887	C	T	0.0189482	0.00277607	8.80E-12	46
20	rs2533273	A	C	−0.00987659	0.00167714	3.90E-09	34
21	rs261809	G	A	−0.00962858	0.00167902	9.80E-09	32
22	rs3101339	C	A	0.0142595	0.00170546	6.20E-17	69
23	rs34162196	T	C	−0.0223628	0.00277168	7.10E-16	65
24	rs3764002	T	C	0.0131215	0.00190081	5.10E-12	47
25	rs4140799	A	G	0.0094566	0.00167846	1.80E-08	31
26	rs4149513	A	G	0.01173	0.00167139	2.20E-12	49
27	rs4269101	G	T	−0.0138099	0.00185921	1.10E-13	55
28	rs429358	C	T	0.0199445	0.00231362	6.70E-18	74
29	rs4800488	A	C	0.0119836	0.00167202	7.70E-13	51
30	rs57499472	C	T	0.00991231	0.00171869	8.10E-09	33
31	rs62084586	C	T	0.0133946	0.00226174	3.20E-09	35
32	rs72720396	G	A	0.0114265	0.00198541	8.70E-09	33
33	rs746868	G	C	−0.0129055	0.00171452	5.20E-14	56
34	rs75641275	C	A	−0.0141613	0.00238518	2.90E-09	35
35	rs7582086	T	G	−0.00962821	0.00167381	8.80E-09	33
36	rs7599488	T	C	−0.0104154	0.00168729	6.70E-10	38
37	rs7808471	C	T	−0.0115362	0.00178604	1.10E-10	41
38	rs7829800	G	A	−0.0104463	0.00178709	5.10E-09	34
39	rs7916868	T	A	0.00960741	0.00167167	9.10E-09	33
40	rs8081370	T	C	−0.0166666	0.00293795	1.40E-08	32
41	rs862227	G	A	−0.0091646	0.00167238	4.30E-08	30
42	rs893856	A	G	−0.013361	0.00234923	1.30E-08	32
43	rs9385269	T	C	0.0120673	0.00168183	7.20E-13	51

GWAS = genome-wide association study, SNP = single nucleotide polymorphism.

### 3.2. Causal relationship between dried fruit intake and meniscal injuries

**Figure 1.**
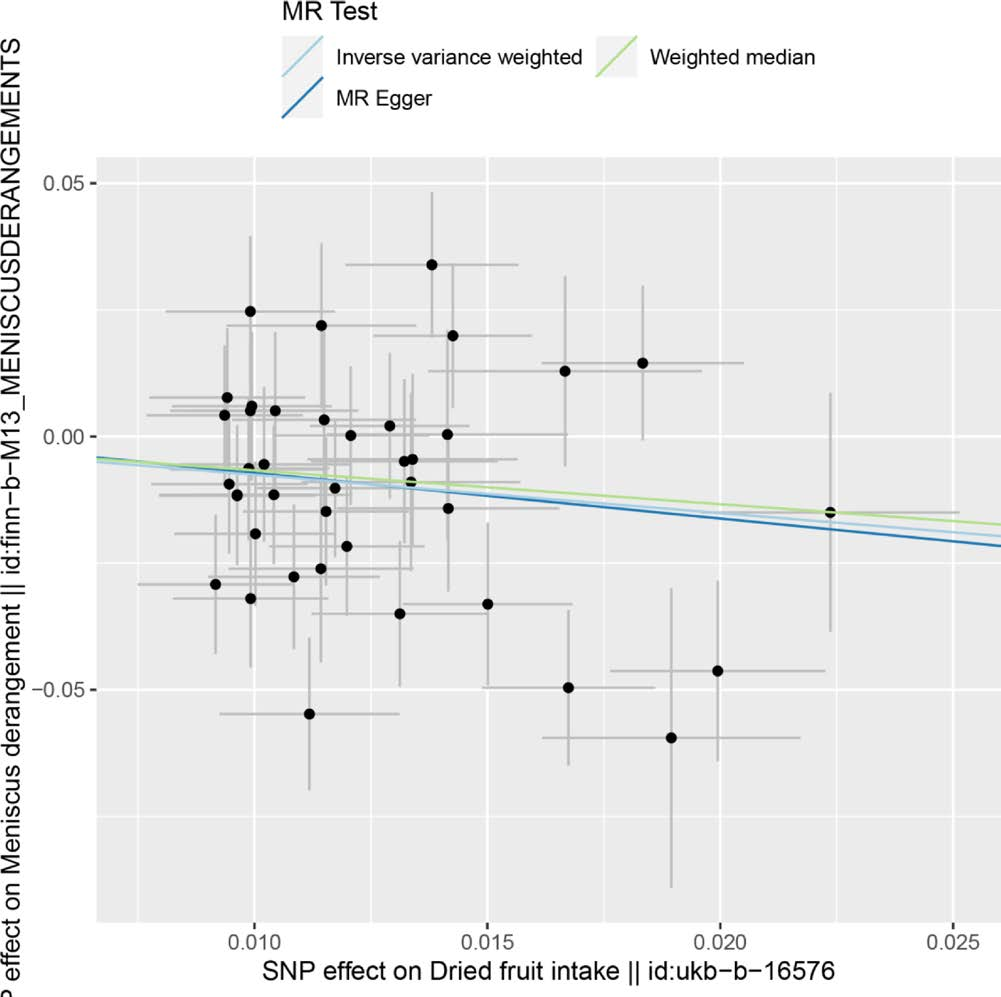
Scatter plot of dried fruit intake and meniscal injuries.

**Figure 2.**
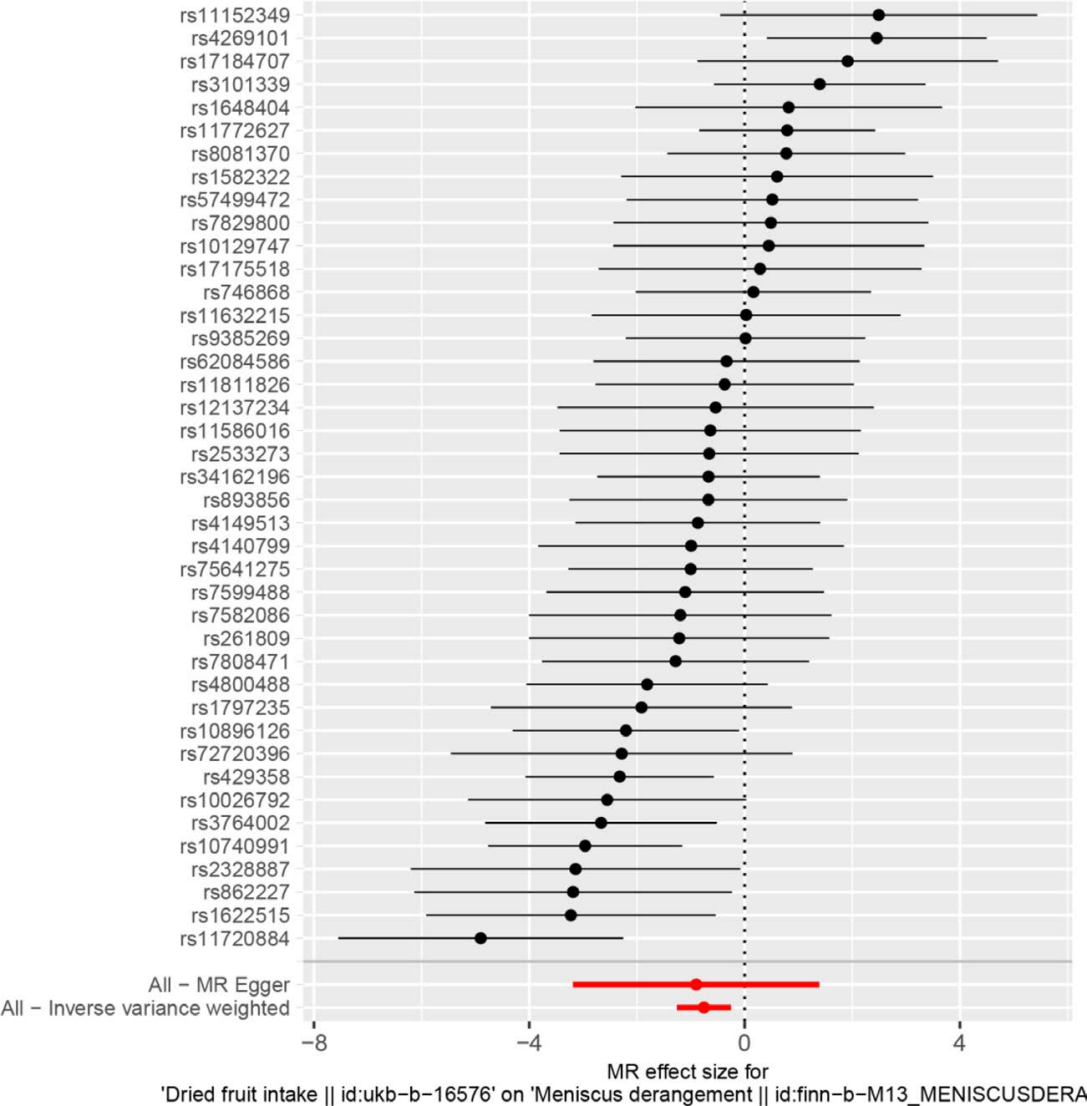
Forest plot of dried fruit intake and meniscal injuries.

By MR analysis, the results of both inverse variance weighting (IVW) and WME showed that there was a causal relationship between dried fruit intake and meniscal injury. IVW: OR = 0.47, 95% CI = 0.28–0.78, *P* = .003; WME:OR = 0.51, 95% CI = 0.28–0.94, *P* = .030; (Table 2). We can see from both the scatter plot (Fig. 1) and the forest plot (Fig. 2) that too little intake of dried fruits increases the risk of developing meniscal injuries.

**Table 2:** MR regression results of the 3 methods.

method	β	SE	OR (95%CI)	*P*
IVW	−0.756	0.257	0.47 (0.28–0.78)	.003
WME	−0.668	0.308	0.51 (0.28–0.94)	.030
MR-Egger	−0.900	0.168	0.41 (0.04–4.01)	.446

IVW = inverse variance weighting, MR = Mendelian randomization, WME = weighted median.

### 3.3. Sensitivity analysis

**Figure 3.**
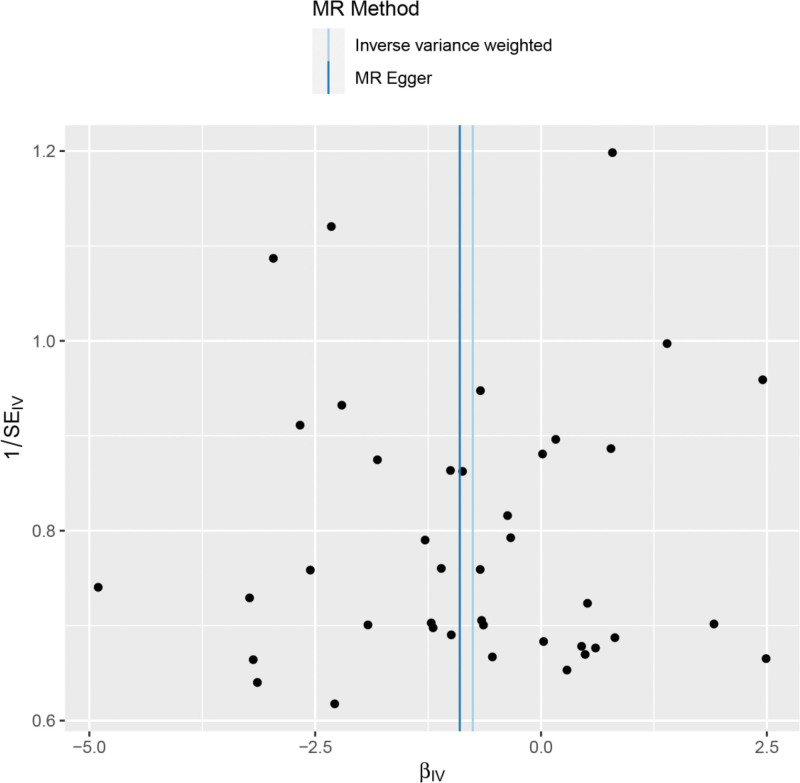
Funnel plot of dried fruit intake and meniscal injuries.

**Figure 4.**
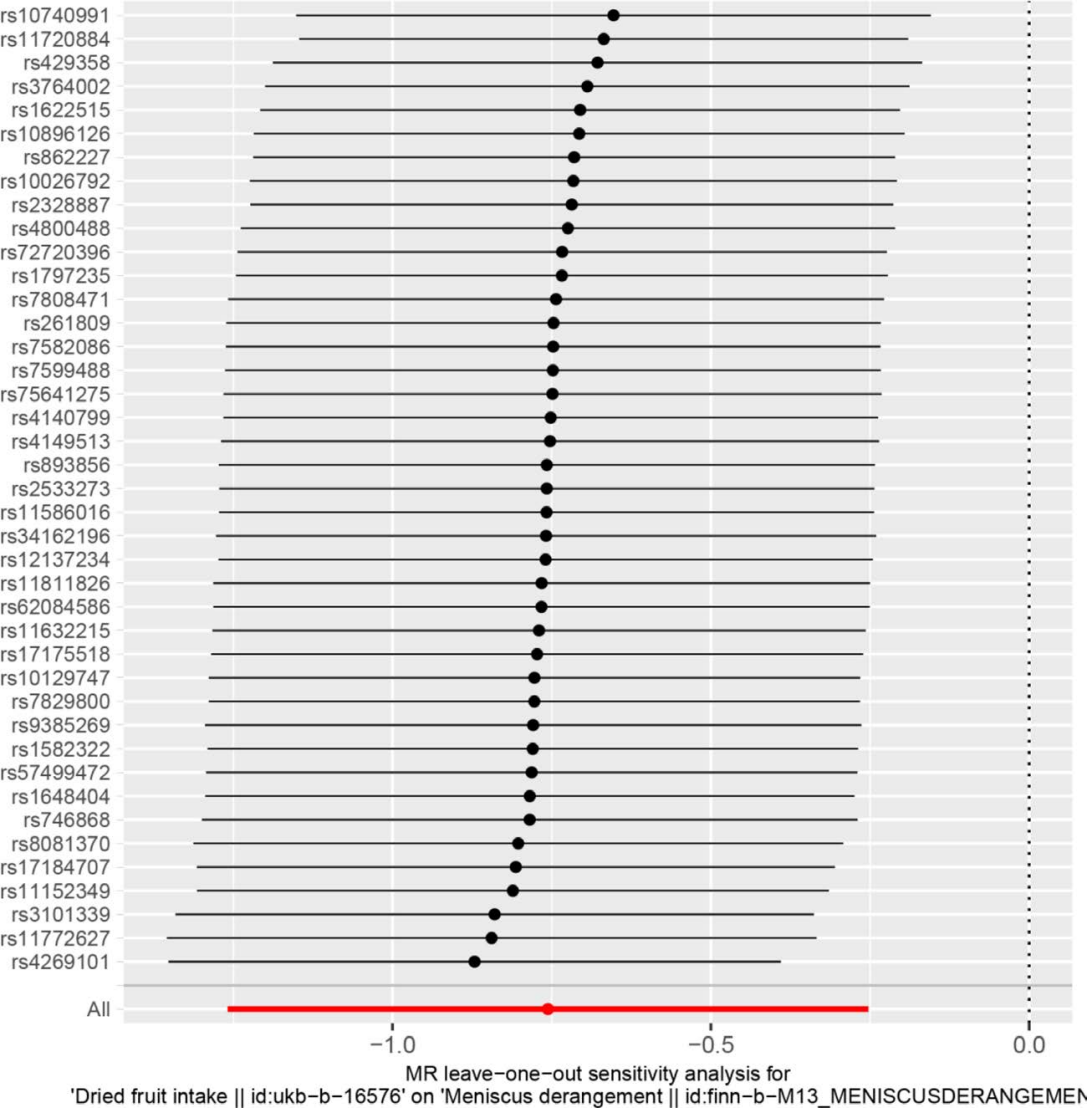
Analysis of dried fruit intake and meniscal injuries by the leave-one-out method.

Heterogeneity was tested using the IVW method (Cochran Q test, *P* = .162) and the results suggested that there was no heterogeneity. A funnel plot was drawn to show the heterogeneity results, as shown in Figure 3. The use of MR-PRESSO was used to screen for SNPs that could lead to heterogeneity, and the results did not reveal any SNPs that would lead to heterogeneity in the results. The result of Global test by MR-PRESSO suggested that there was no pleiotropy (*P* = .900). The “leave-one-out” method uses the IVW method by default, and as can be seen in Figure 4, no single SNP will have a large impact on the overall results after eliminating any SNP, indicating that the results are robust.

## 4. Discussion

It is known that an excessively low intake of dried fruit may be an observational risk factor for meniscal injuries, but the causality of this association is unclear. Our MR study aimed to reveal the causal relationship between dried fruit intake and meniscal injury. The results of the 2-sample MR showed that there was a causal association between dried fruit intake and meniscal injury with an OR (95 % CI) of 0.47 (0.28–0.78), *P* = .003, suggesting that those with excessively low intake of dried fruits had a higher risk of meniscal injury compared to the general population.

The incidence of meniscal injuries is reported to be increasing.^[20]^ Conservative and surgical treatments are commonly used.^[21]^ For some minor meniscus injuries, conservative treatment methods can be used, including rest, cold packs, pain medications, and physical therapy.^[22]^ These methods can help reduce pain and inflammation and promote self-healing of meniscal injuries. For severe meniscal injuries, such as those with large meniscal tears or those that result in limited joint function, surgical treatment may be required.^[23]^ Surgery can repair or remove damaged meniscal tissue to restore normal joint function. There are many factors that influence meniscal injuries,^[24]^ and knowing the risk factors for meniscal injuries is important for clinical practice. Knowing the risk factors for meniscal injuries can help physicians to risk assess patients and develop individualized treatment plans. Previous studies have reported several risk factors for meniscal injuries, including age, male gender, type of physical activity, higher body mass index (BMI), and delayed repair of concomitant anterior cruciate ligament (ACL) injuries.^[25–29]^ However, there is a lack of research on the effect of dried fruit intake on meniscal injuries. Previous studies have reported that too little dry fruit intake is a risk factor for many diseases such as cardiovascular disease, asthma and low back pain.^[5–7]^ Similarly, the present study confirmed that dry fruit intake was negatively associated with the risk of meniscal injury from a genetic perspective. In addition, statistical evidence from sensitivity analyses strongly supports our findings. Therefore, awareness of the dangers of too little dried fruit intake should be increased. Screening for meniscal injuries should be increased in people with low intake of dried fruits, so that patients with meniscal injuries can be detected and treated in a timely manner, which is beneficial to the prognosis of the patients. For patients with meniscal injuries, increased intake of dried fruits should be advocated.

The mechanism underlying the causal relationship between dried fruit intake and meniscal injuries is unclear. Dried fruits are obtained from fresh fruits by using various drying techniques. They are important healthy snacks and are a rich source of dietary fiber, minerals, vitamins and various bioactive compounds such as flavonoids and carotenoids.^[30]^ Dried fruits exert a variety of biological effects, including antioxidant, anti-inflammatory, anti-atherosclerotic and anticancer effects.^[31,32]^ Experimental studies have shown that dried fruit intake inhibits pro-inflammatory cytokines and promotes the function of the musculoskeletal system.^[33,34]^ In clinical studies, many authors have found that daily intake of dried fruits has a protective effect on musculoskeletal health in both men and women.^[35,36]^ However, further experimental and clinical studies are needed due to limited evidence on the underlying mechanisms

There are also some limitations of this study. Firstly, as all data are from people of European origin, the results are not representative of a truly randomized population sample and are not applicable to other so races. Second, although various sensitivity analyses have been performed in this study to test the hypotheses of the MR study, it is also difficult to completely rule out horizontal pleiotropy of instrumental variables. Finally, the current sample size of GWAS data is still not large enough, and more in-depth studies using more GWAS data are needed in the future.

## 5. Conclusion

In conclusion, this study used a 2-sample MR analysis to analyze and explore the genetic data, which showed that too little dried fruit intake is a risk factor for developing meniscal injuries.

## Author contributions

**Conceptualization:** Guang-Hua Deng.

**Data curation:** Guang-Hua Deng.

**Formal analysis:** Guang-Hua Deng.

**Funding acquisition:** Guang-Hua Deng.

**Investigation:** Guang-Hua Deng.

**Methodology:** Guang-Hua Deng.

**Project administration:** Guang-Hua Deng.

**Resources:** Guang-Hua Deng.

**Software:** Guang-Hua Deng.

**Supervision:** Guang-Hua Deng.

**Validation:** Guang-Hua Deng.

**Visualization:** Guang-Hua Deng.

**Writing – original draft:** Guang-Hua Deng.

**Writing – review & editing:** Guang-Hua Deng.
